# Spatial disparities and associated factors of composite index of anthropometric failure for under-five children across three African countries

**DOI:** 10.1016/j.gloepi.2026.100268

**Published:** 2026-05-25

**Authors:** Nigussie Adam Birhan, Abebew Aklog Asmare, Kefale Tilahun Getahun, Zelalem Meraf wolde, Denekew Bitew Belay, Ding-Geng Chen

**Affiliations:** a Department of Statistics, College of Natural and Computational Science, Injibara University, Injibara, Ethiopia; b Department of Statistics, College of Natural and Computational Science, Mekdela Amba University, Gimba, Ethiopia; c Department of Statistics, College of Science, Bahir Dar University, Bahir Dar, Ethiopia; d School of Health Systems and Public Health, Faculty of Health Sciences, University of Pretoria, Pretoria, South Africa; e Department of Statistics, University of Pretoria, Pretoria, South Africa; f Arizona State University, College of Health Solution, Phoenix, AZ, USA

**Keywords:** Associated factors, Composite index of anthropometric failure, Bayesian geo-additive model, Provinces, Spatial variation

## Abstract

**Background:**

Malnutrition is associated with both undernutrition and over nutrition. Child undernutrition remains the greatest public health problem in developing countries. This study assessed factors associated with the Composite Index of Anthropometric Failure (CIAF) and explored spatial patterns across the provinces of three African countries.

**Methods:**

A nationally representative secondary record from the recent Demographic and Health Survey was used. Spatial Autocorrelation was used to identify particular provinces clustering with high and low CIAF values. A Bayesian generalized geo-additive mixed modelling approach was applied using Integrated Nested Laplace Approximation with a binomial family and logit link function.

**Results:**

The overall prevalence of CIAF among the three African countries was 41.2%, with a difference between countries ranging from Zambia (34.9%) to Democratic Republic of Congo (46.0%). Both Moran's I and Geary's C tests evidenced the existence of spatial autocorrelation of CIAF among children in the three African countries. Bayesian generalized geo-additive model with Besag-York-Mollie mixed effect was found to be the best model to assess the spatial dependencies and the non-linear effects of factors on CIAF. This study showed the existence of spatial disparities in the CIAF. Place of residence, mother's education level, child sex, wealth index, marital status, access to health facilities, child birth size, type of birth, diarrhea, vitamin A supplementation, place of birth, child anemia status, iron supplementation, and country were associated with CIAF.

**Conclusion:**

This study revealed a high burden of CIAF across the three African countries, with marked spatial disparities and clustering of CIAF in children. The Bayesian geo-additive model identified multiple socioeconomic, demographic, geographical, and health-related factors associated with the CIAF. These findings highlight the need for geographically targeted, multifaceted interventions to address the underlying factors associated with malnutrition and reduce regional inequalities.

## Introduction

Child malnutrition is a major public health problem worldwide, with an estimated 45% of all deaths among children under five years of age [1]. Child undernutrition is the most common form of malnutrition and a major contributor to child mortality in low- and middle-income countries [2], [3]. It is a major public health problem with major consequences for child survival, growth, mortality, cognitive ability, and psychosocial development, as well as the economic productivity of individuals and societies [4], [5], [6], [7], [8], [9].

Child undernutrition is a major public health issue in developing countries, ranked as a top global challenge with severe human and economic impacts, especially on the poor, women, and children [10], [11], [12]. In 2022, an estimated 149 million children under five years of age worldwide were stunted, and 45 million were wasted [13]. In sub-Saharan Africa, malnutrition has increased from 5.5 million to 30 million in the past decade, contributing to more than 3.5 million deaths of children under five years of age annually [14], [15]. The region bears the highest burden of undernutrition compared to other sub-regions due to poverty, food insecurity, political instability, climate variability, inadequate infrastructure, and poor feeding practices [16], [17], [18].

Previous studies on the prevalence of undernutrition and its associated factors have typically focused on a specific indicator, such as stunting, wasting, and underweight using conventional nutritional assessment methods [19], [20], [21], [22]. However, underweight children may experience stunting and/or wasting, and some children may suffer all three anthropometric failures simultaneously. Therefore, none of these conventional nutritional indicators can precisely characterize the overall burden of under-nutrition among under five children. To address this issue, the composite index of anthropometric failure, a multidimensional single index, was introduced by Svedburg and Nandy in 2000, which may show signs of two or more anthropometric failures simultaneously [23], [24].

The CIAF offers a comprehensive view of childhood undernutrition in resource-limited settings by combining various categories of anthropometric failures, such as wasting only, wasting and underweight, wasting, stunting, and underweight, stunting and underweight, stunting only, and underweight only. It is widely recommended as a reliable measure of malnutrition, helping identify multiple nutritional deficiencies through a single aggregated indicator [24], [25], [26]. The literature supports the use of CIAF rather than traditional methods for assessing the nutritional status of children [27].

Despite its importance, CIAF has been insufficiently studied in the Democratic Republic Congo (DRC), Zambia, and Angola, three neighboring countries that continue to experience high levels of food insecurity, poverty, conflict-related disruptions, and limited access to health and nutrition services. These countries have some of the highest child malnutrition rates in Africa; however, the spatial distribution of the CIAF and its underlying determinants remain poorly understood [28], [29]. Furthermore, malnutrition in these settings is not uniformly distributed; it varies across provinces due to differences in socioeconomic conditions, environmental factors, health service availability, and household characteristics.

This study provides new estimates for the prevalence of undernutrition by aggregating traditional undernutrition indices, which are important for capturing the overall impact of undernutrition on a population, unlike any of the three traditional indicators. Addressing spatial disparities in the CIAF in the provinces requires a multifaceted approach targeting socioeconomic, geographical, and health system barriers. As geographic disparities in healthcare services pose challenges for policymakers and practitioners, assessing the geographical pattern of CIAF using the most recent Demographic and Health Survey (DHS) data is critical for planning and implementing geographically focused interventions to reduce the persistent burden of childhood anthropometric failure across the three countries. Therefore, this study aimed to analyse the spatial distribution with contributions to understand disparities in the CIAF and associated factors among children in the three Africa countries.

## Materials and methods

### Data sources and study area

**Fig. 1:**
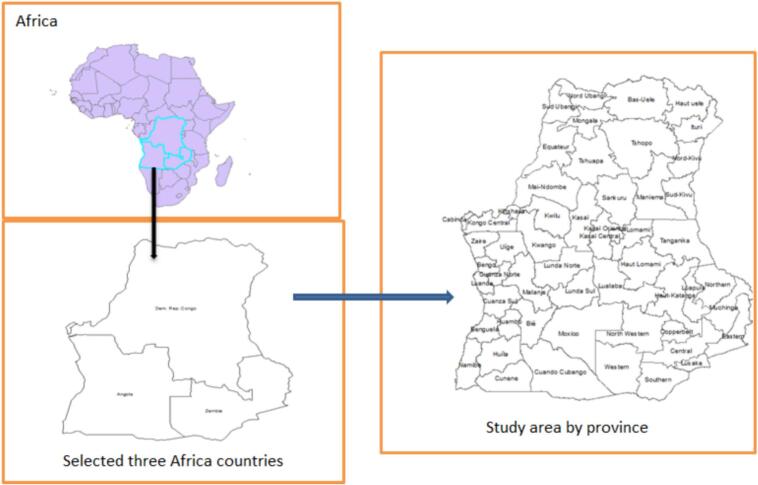
Countries considered in this study.

The data used in this study were obtained from a nationally representative Demographic and Health Survey (DHS) conducted in three African countries between 2023 and 2024. The data is freely available upon reasonable request and can be accessed from https://dhsprogram.com/. The shape files for the administrative provinces of each country are also obtained from https://www.diva-gis.org/gdata. We used Kid Record (KR) files to extract the study participants. The current analysis included children who were live births in the five years preceding the time of the interview and who participated in the surveys in three African countries (Fig. 1). All DHS surveys employ a multistage stratified cluster sampling technique. In the first stage, enumeration areas (clusters) are selected using probability proportional to size, followed by systematic sampling of households within each cluster. Sampling weights, primary sampling units and stratification variables are provided in the DHS datasets to ensure representativeness at national and subnational levels. For the pooled analysis, datasets from the three countries were combined after harmonizing variable definitions and coding schemes. The DHS sampling weights were applied and further de-normalized and rescaled to account for differences in sample size and population structure across countries, ensuring appropriate contribution of each country. All records related to malnutrition with complete records in all DHS documents were included in the study. A total of 13,607 children were included in this study. A multistage stratified sampling technique was used for all demographic and health survey data in low-income countries.

#### Study variables

In this study, the outcome variable was the CIAF among children coded as 1 = failures, 0 = not a failure. Children who did not show any signs of anthropometric failure from categories B to Y were classified as “no failure”. The purpose of the CIAF is to identify all undernourished children and measure the total burden of child malnutrition on the population. The CIAF helps disaggregate malnourished children into different subgroups. B, F, and Y are groups with single-burden anthropometric failure. E and C are groups with double burden of anthropometric failure. Group D was the group with triple-burden anthropometric failure. According to CIAF standards, a child was classified as having “anthropometric failure” if the child exhibited any of the deficiencies listed in categories B to Y as it is clearly depicted in (Table 1).

**Table 1:** Category of anthropometric failure in under five children using Composite Index of Anthropometric (CIAF).

Groups	CIAF categories	Description of the level	Wasting	Stunting	Underweight
A	No failure	Normal WAZ, HAZ and WHZ	No	No	No
B	Wasting only	WHZ < -2SD, but normal HAZ and WAZ	Yes	No	No
C	Wasting, underweight	WHZ and WAZ < -2SD, but HAZ normal	Yes	No	Yes
D	Wasting, stunting and underweight	WHZ, HAZ and WAZ < -2SD	Yes	Yes	Yes
E	Stunting, underweight	HAZ and WAZ < -2SD, but WHZ normal	No	Yes	Yes
F	Stunting only	HAZ < -2SD, but normal WAZ and WHZ	No	Yes	No
Y	Underweight only	WAZ < -2SD, but normal HAZ and WHZ	No	No	Yes

#### Independent variables

**Fig. 2:**
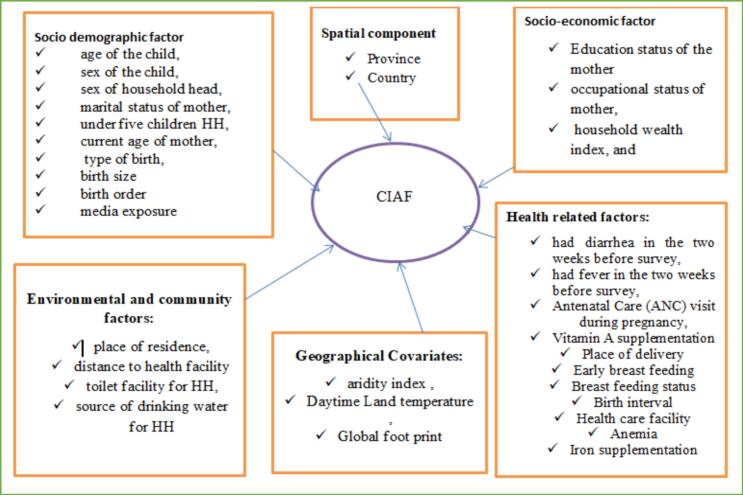
Covariates included in the model.

Our selection of these variables was informed by the United Nations International Children's Emergency Fund conceptual framework of the factors contributing to undernutrition [20]. Furthermore, the availability of potential associated factors in the DHS dataset was considered while choosing the explanatory variables (Fig. 2).

### Statistical model and data analysis

#### Data explorations and extraction

Data extraction and descriptive statistics were performed using STATA version 18. In the descriptive analysis, frequencies and percentages were explored using tables and figures. We first apply a linear model and extend it by relaxing the effect of the risk factor to see the parsimonious estimation of each parameter. We then extended the linear model to an additive fixed effect to observe its improvements in predicting the CIAF. We also extended the fixed effects additive model to an additive mixed model. Moreover, we tested the existence of spatial autocorrelation using different spatial dependency tests and province structure approaches. Finally, by considering the existence of both additive mixed effects of the risk factors on the CIAF and the existence of spatial autocorrelation, we applied a geo-additive modelling approach to fit the data to fully leverage these two data properties in our model [30], [31], [32], [33].

#### Spatial autocorrelation

Spatial autocorrelation measures the degree to which a variable or phenomenon is correlated with itself based on its spatial location. It evaluates the orientation and strength of the relationship between a variable and its province values. Global spatial autocorrelation is defined as a measure of the overall clustering of data, which provides a correlation statistic to summarize the entire study area [34]. In this study, we used Global Moran's I and Geary's C statistics to evaluate the spatial dependency of the CIAF of children across three countries. The calculated Moran's I values close to −1 indicate that it is dispersed, whereas close to +1 indicates that it is clustered and is distributed randomly if the value is zero [34], [35], [36]. Geary's C is another global measure of spatial autocorrelation. Unlike Moran's I, it is more sensitive to local variations rather than overall patterns, emphasizing differences between province values. Geary's C > 1 indicates negative spatial autocorrelation (dissimilar values are clustered), Geary's C < 1 indicates positive spatial autocorrelation (similar values are clustered), and Geary's C = 1 indicates no spatial autocorrelation (random spatial pattern) [37]. This study employed a hierarchical Bayesian framework to model the probability of CIAF in three African countries. Three progressively complex models were estimated and compared: the generalized linear mixed model (GLMM), generalized additive mixed model (GAMM), and generalized geo-additive mixed model (GGAMM).

#### Generalized geo-additive model

To assess the associated factors on the CIAF, we adopted a geo-additive model using a Bayesian approach. This approach allows for the simultaneous estimation of the spatial effects of geographical locations, the unknown nonlinear effect of covariates, and the vectors of fixed effect parameters. This approach also provides monotonicity constraint estimates and allows borrowing extensions developed for Bayesian mean regression, such as multilevel structures and regularization priors, without the necessity to redevelop the complete inferential machinery [37], [38], [39].

Integrated Nested Laplace Approximation (INLA) is a computational method for Bayesian inference in latent Gaussian models (like spatial models), offering a fast and accurate alternative to traditional Markov Chain Monte Carlo (MCMC) methods. Unlike MCMC, which focuses on estimating the highly multivariate joint posterior distribution, INLA estimates the univariate marginal posterior distribution of latent fields and hyper parameters. INLA's computational efficiency and superiority of INLA stem from its use of sparse representations of high-dimensional precision matrices in latent Gaussian models and its combination of Laplace approximations and nested integration strategies. These advantages make INLA widely applicable in various fields, and it has been implemented in the R-INLA package [40], [41], [42].

In our analysis, the outcome is binary and was modelled using a binomial distribution with a logit link. The model for i^th^ child p province can be written as.

$$\text{logit}\left(\mathrm{Pr}\left(y_{i}=1\right)\right)=Z_{i}\beta +\sum_{j=1}^{J}f_{j}\left(x_{\mathrm{ji}}\right)+f_{\mathrm{str}}\left(p\right)+f_{\text{unst}}\left(p\right)$$

where $Z_{i}$ is the row vector of fixed-effect covariates and β is the corresponding vector of coefficients. The functions $f_{j}\left(x_{\mathrm{ji}}\right)$ represent smooth nonlinear effects of continuous covariates. The structured spatial effect $f_{\mathrm{str}}\left(p\right)\;$is specified at the province level and modelled using an intrinsic conditional autoregressive prior to capture spatial dependence between neighboring provinces, while the unstructured spatial effect $f_{\text{unst}}\left(p\right)$ is modelled as independent Gaussian noise. In INLA, the structured spatial effect has precision $\tau_{u}$ and the unstructured spatial effect has precision $\tau_{v}$. Each child-level observation inherits the spatial effect of the province in which it resides, allowing the model to account for correlations between neighboring provinces.

### Model performance and validation

Model comparison was performed using conventional Bayesian fit metrics, such as the Deviance Information Criterion (DIC), Watanabe-Akaike Information Criterion (WAIC), and Conditional Predictive Ordinate (CPO) [41], [43].

The model with the lowest DIC and WAIC values and the highest CPO was selected as the best fit. The DHS sampling weights were used to account for differential selection probability and non-response, guaranteeing population representativeness across the three African countries. Weights were added using the weights option in the INLA function. The test statistic, such as the Area under the Curve (AUC), which is a function of sensitivity and specificity, is used to assess the overall prediction abilities of the selected model.

## Result

### Descriptive results

Descriptive statistics for each country, survey year (data collection year), distribution of CIAF by country, and the proportion of CIAF among the three African countries were reported. A total of 13,607 under five children were included in the study. The pooled proportion of CIAF in three African countries was 41.2% (95%CI: 40.4, 42.1), with difference compared with country-specific proportions, which ranged from 34.9% in Zambia to 46.0% in the DRC (Table 2).

**Table 2:** Proportion of under five children who had CIAF by country, province, survey year, and overall proportion across countries in Africa.

Country	Survey year	Survey sample	Number of Province	Number of CIAF cases	Percentage with CIAF	95%CI
Angola	2023	2893	18	1205	41.7	39.9,4 3.5
DRC	2023	6043	26	2777	46.0	44.7, 47.2
Zambia	2024	4671	10	1630	34.9	33.5, 36.3
Overall		13,607	54	5612	41.2	40.4, 42.1

### Characteristics of covariate variables

Among women in this study, 15.9% of women had no formal education, 19.7% had no ANC, and 47.3% had no working. Of the total study participants, 6.2% had never been breastfed, and 78.5% had received iron supplementation. Most independent variables were associated with CIAF, except sex of the household head and early breastfeeding (Table 3).

**Table 3:** Distributions of independent variable and their association with CIAF among 3 African countries.

Variables	Categories	No CIAF (%)	CIAF (%)	Total (%)	Chi-square *p*-value
Place of residence	Urban	3601(45.0)	1697(30.2)	5298(38.9)	<0.001
	Rural	4394(55)	3915(69.8)	8309(61.1)	
Level of maternal education	No education	1051(13.2)	1109(19.8)	2160(15.9)	<0.001
	Primary education	2509(31.4)	2155(38.4)	4664(34.3)	
	Secondary and higher education	4435(55.5)	2348(41.8)	6783(49.9)	
Birth size of the child	Small	505(6.3)	579(10.3)	1084(8.0)	<0.001
	Average	4462(55.8)	3249(57.9)	1711(56.7)	
	Large	3029(37.9)	1783(31.8)	4812(35.4)	
Diarrhea	No	6654(83.2)	4475(79.7)	11,129(81.8)	0.002
	Yes	1341(16.8)	1137(20.3)	2478(18.2)	
Access to health facility	Not a big problem	3595(45.0)	2047(36.5)	5642(41.5)	<0.001
	Big problem	4400(55.0)	3565(63.5)	7965(58.5)	
Fever	No	6511(81.4)	4407(78.5)	10,918(80.2)	<0.001
	Yes	1484(18.6)	1205(21.5)	2689(19.8)	
Drinking water source	Not improved	3278(41.0)	2872(51.2)	6150(45.2)	<0.001
	Improved	4717(59.0)	2740(48.8)	7457(54.8)	
Mother's occupation	Not working	3973(49.7)	2467(44.0)	6440(47.3)	<0.001
	Working	4022(50.3)	3145(56.0)	7167(52.7)	
Type of birth	Single	7832(98.0)	5313(94.7)	13,145(96.6)	<0.001
	Multiple	163(2.0)	299(5.3)	462(4.0)	
Sex of the child	Male	3749(46.9)	3124(55.7)	6972(50.5)	<0.001
	Female	4247(53.1)	2488(44.3)	6735(49.5)	
Toilet facility	Not improved	1511(18.9)	1354(24.1)	2865(21.1)	<0.001
	Improved	6485(81.1)	4258(75.8)	10,742(79.0)	
Child anemia status	Not anemic	2164(27.1)	1544(27.5)	3708(27.3)	<0.001
	Mild	1742(21.8)	1381(24.6)	3123(23.0)	
	Moderate	4002(50.1)	2543(45.3)	6545(48.1)	
	Severe	87(1.1)	144(2.6)	231(1.7)	
Wealth index	Poor	3263(40.8)	2984(53.2)	6247(45.9)	<0.001
	Middle	1547(19.4)	1235(22.0)	2782(20.4)	
	Rich	3185(39.8)	1393(24.8)	4578(33.7)	
Media exposure	No	3735(46.7)	3294(58.7)	7028(51.7)	<0.001
	Yes	4260(53.3)	2318(41.3)	6579(48.4)	
Birth order of the child	First	1896(23.7)	1246(22.2)	3142(23.1)	<0.001
	2 to 3	2785(34.84	1930(34.4)	4716(34.7)	
	4 to 5	1834(22.9)	1309(23.3)	3143(23.1)	
	6 and more	1480(18.5)	1126(20.1)	2606(19.2)	
Iron supplementation	No	1527(19.1)	1406(25.1)	2933(21.6)	<0.001
	Yes	6468(80.9)	4206(75.0)	10,674(78.5)	
Birth interval	First	1902(23.8)	1255(22.4)	3157(23.2)	<0.001
	Less than 33 months	2411(30.2)	2045(36.4)	4455(32.7)	
	33–59 months	2487(31.1)	1633(29.1)	4120(30.3)	
	Above 59 months	1196(14.9)	679(12.1)	1875(13.8)	
Sex of household head	Male	5754(72.0)	4145(73.8)	9899(72.6)	**0.9**
	Female	2241(28.0)	1466(26.1)	37,708(27.3)	
Breastfeeding status	Ever breastfed	2413(30.2)	2279(40.6)	4691(34.5)	<0.001
	Never breastfed	416(5.2)	421(7.5)	837(6.2)	
	Still breastfeeding	5167(64.6)	2912(51.9)	8079(59.4)	
Place of delivery	Home	1278(16.0)	1219(21.7)	2497(18.4)	<0.001
	Health institution	6717(84.0)	4393(78.3)	11,110(81.7)	
Vitamin A supplementation	No	4369(54.7)	3030(54.0)	7399(54.4)	**0.1**
	Yes	3626(45.4)	2582(46.0)	6208(45.6)	
Marital status	Unmarried	890(11.1)	581(10.4)	1471(10.8)	<0.001
	Married	4359(54.5)	2772(49.4)	7131(52.4)	
	Separated	2746(34.4)	2259(40.3)	5005(36,8)	
ANC	No	752(9.4)	707(12.6)	1459(19.7)	<0.001
	1–7	6903(86.3)	4755(84.7)	11,658(85.7)	
	8 and above	340(4.3)	149(2.7)	490(3.6)	
Early breast feeding	No	2776(34.7)	1975(35.2)	8856(65.1)	**0.5**
	Yes	5220(65.3)	3636(64.8)	4751(34.9)	

### Spatial autocorrelation analysis

**Fig. 3:**
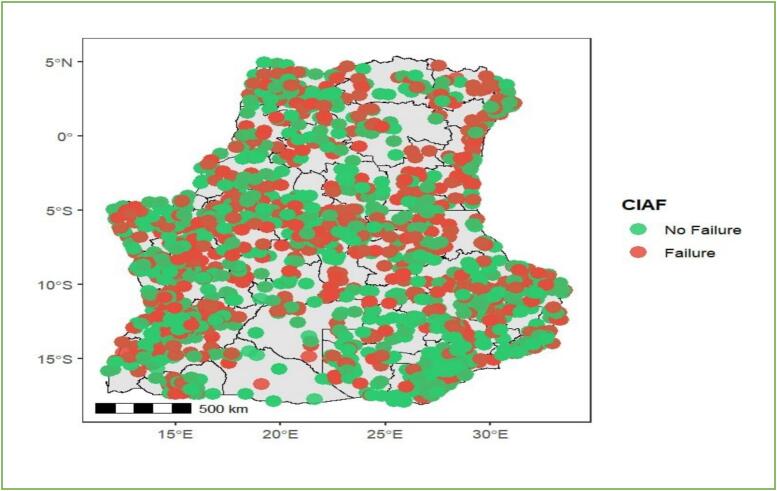
Spatial autocorrelation test for CAIF.

The spatial variation in CIAF is depicted descriptively in Fig. 3, with provinces classified according to no failure and failure. This mapping highlights notable geographic disparities to formally assess spatial autocorrelation using global Moran's I and Geary's C The estimated Moran's I and Geary's C values were 0.3 and 0.7, respectively. Moran's I indicates overall (global) spatial autocorrelation, whereas Geary's C is more sensitive to local spatial variation. Values of Geary's C less than 1 suggest positive spatial autocorrelation which provide evidence that similar CIAF values tend to cluster geographically.

#### Model fit comparison for linear versus nonlinear covariate effects

We assessed whether temperature, child age, and the number of under-5 children were better represented as linear terms or nonlinear smooth functions. Hence, the model with nonlinear specifications showed a better fit, with lower values of the DIC = 16,024 and WAIC = 16,395 compared with DIC = 16, 398 and WAIC = 16, 506 indicate improved model performance Based on these findings, the nonlinear specification was retained in the main analysis to better capture potential nonlinear relationships between these covariates and the outcome.

**Fig. 4:**
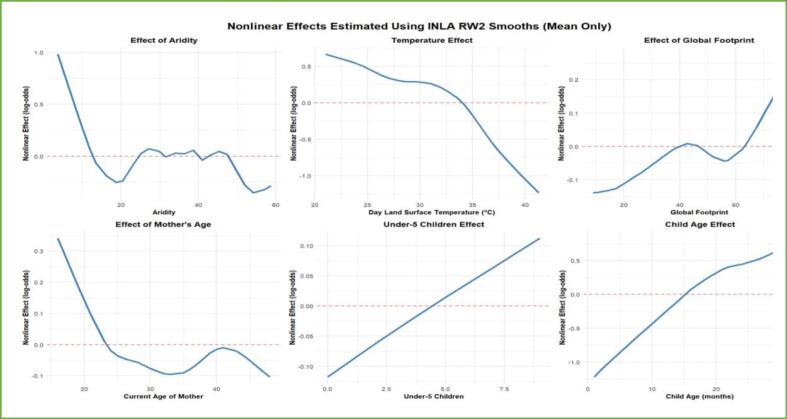
Plots of the posterior mean of the non-linear effect of risk factors.

For other nonlinear risk factors, the effects of these factors on the CIAF were estimated using an additive assumption. The effects of these random factors, as shown in Fig. 4, indicate non-linear effects. The posterior mean effects depicted in both the figures and the table for all non-linear risk factors have shown their effect with their 95% credible interval.

One of the factors with a non-linear effect is the mother's current age, which shows a decreasing effect, indicating that as the age of the mother increases, the CIAF decreases. This might be due to the fact that older mothers might have experience in child caring. Similarly, as the age of the child increased, the CIAF increased. The number of under-five children also had a positive effect on the CIAF, which also had an increasing effect. The other non-linear association was observed between environmental factors and CIAF: aridity, day Land Surface Temperature and Global Footprint extremes. Children in areas with very low aridity are at a higher risk of CIAF, but the effect diminishes as aridity increases.

#### Spatial effects

**Fig. 5:**
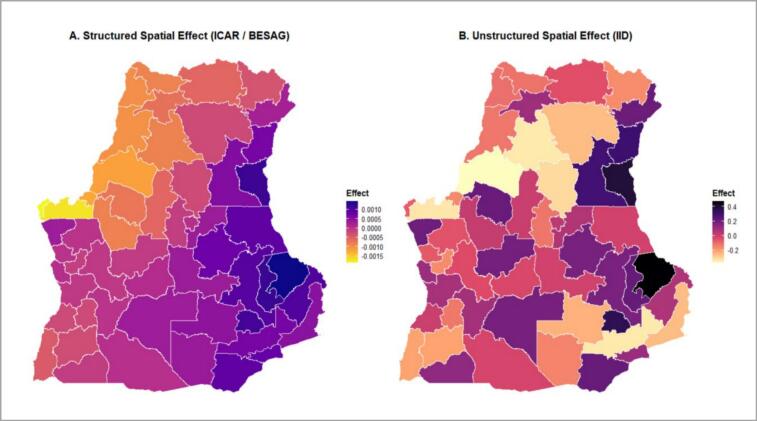
Posterior means of structured (A) and unstructured (B) spatial effects on CIAF in three African countries.

The posterior mean estimates of the structured and unstructured spatial effects across provinces in the three African countries reveal two unique trends. The structural component (Panel A) demonstrates a clear geographic grouping, indicating that the surrounding provinces have similar CIAF results. In contrast, the unstructured component (Panel B) represents isolated deviations at the provincial level, demonstrating regional heterogeneity. These trends demonstrate both regional homogeneity and province-specific heterogeneity in the CIAF results (Fig. 5).

**Fig. 6:**
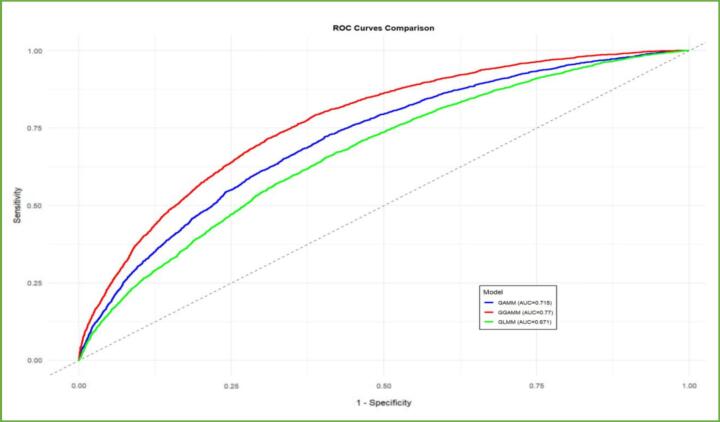
Roc curve for model comparison.

Fig. 6 shows the ROC comparison, which shows that the GGAMM model provides the highest in sample discrimination, with an AUC of 0.771, among the compared models GAMM (AUC = 0.715) and GLMM (AUC = 0.671).

The model comparison using different information criteria were performed. Among the fitted models, the GGAMM was the best-performing model, with the lowest DIC and WAIC and the highest CPO, indicating good apparent discrimination within the study sample and overall model fit (S1).

### Fixed effect analysis

In this study, place of residence, level of education, sex of the child, wealth index, marital status, access to health facilities, birth size of the child, type of birth, diarrhea, vitamin A supplementation, place of birth, child anemia status, iron supplementation, and country were associated factors of CIAF among the three African countries (Table 4).

**Table 4:** Estimated posterior means of the parameter with its corresponding 95% credible intervals.

Factors (fixed effects)	Total (%)	aOR (95% CrI)
Drinking water source		
Not improved (ref)	6150 (45.2)	1
Improved	7457 (54.8)	0.97 (0.88, 1.08)

Breastfeeding status		
Ever breastfed (ref)	4691 (34.5)	1
Never breastfed	837 (6.2)	0.91 (0.77, 1.08)
Still breastfeeding	8079 (59.4)	1.03 (0.90, 1.18)

Place of residence		
Urban (ref)	5298 (38.9)	1
Rural	8309 (61.1)	1.26 (1.08, 1.46)

Level of maternal education		
No education (ref)	2160 (15.9)	1
Primary education	4664 (34.3)	1.02 (0.90, 1.15)
Secondary and higher	6783 (49.9)	0.73 (0.64, 0.83)

Sex of child		
Male (ref)	6972 (50.5)	1
Female	6735 (49.5)	0.62 (0.57, 0.67)

Wealth index		
Poor (ref)	6247 (45.9)	1
Middle	2782 (20.4)	0.92 (0.82, 1.04)
Rich	4578 (33.7)	0.66 (0.57, 0.80)

Marital status		
Unmarried (ref)	1471 (10.8)	1
Married	7131 (52.4)	0.85 (0.73, 0.99)
Separated	5005 (36.8)	0.98 (0.84, 1.14)

Access to health facility		
Not a big problem (ref)	5642 (41.5)	1
Big problem	7965 (58.5)	1.13 (1.04, 1.24)

Toilet facility		
Not improved (ref)	2865 (21.1)	1
Improved	10,742 (78.9)	1.03 (0.93, 1.15)

Birth size of the child		
Small (ref)	1084 (8.0)	1
Average	7711 (56.7)	0.60 (0.52, 0.70)
Large	4812 (35.4)	0.41 (0.35, 0.48)

Type of birth		
Single (ref)	13,145 (96.6)	1
Multiple	462 (3.4)	2.84 (2.24, 3.61)

Diarrhea		
No (ref)	11,129 (81.8)	1
Yes	2478 (18.2)	1.28 (1.15, 1.42)

Fever		
No (ref)	10,918 (80.2)	1
Yes	2689 (19.8)	0.99 (0.89, 1.09)

Maternal occupation		
Not working (ref)	6440 (47.3)	1
Working	7167 (52.7)	1.04 (0.95, 1.13)

Vitamin A supplementation		
No (ref)	7399 (54.4)	1
Yes	6208 (45.6)	0.91 (0.83, 0.99)

Place of delivery		
Home (ref)	2497(18.4)	1
Health institution	11,110(81.6)	0.85 (0.75, 0.97)

Birth interval		
First (ref)	3157 (23.2)	1
<33 months	4455 (32.7)	1.42 (0.44, 4.54)
33–59 months	4120 (30.3)	1.22 (0.38, 3.90)
≥59 months	1875 (13.8)	1.27 (0.40, 4.08)

Child anemia		
Not anemic (ref)	3708 (27.3)	1
Moderate	6545 (48.1)	1.14 (1.02, 1.27)
Mild	3123 (23.0)	1.33 (1.20, 1.48)
Severe	231 (1.7)	1.95 (1.43, 2.66)

ANC visits		
No (ref)	1459 (10.7)	1
1–7 visits	11,658 (85.7)	1.11 (0.95, 1.29)
≥8 visits	490 (3.6)	0.83 (0.64, 1.10)

Media exposure		
No (ref)	7028 (51.7)	1
Yes	6579 (48.3)	0.95 (0.86, 1.04)

Iron supplementation		
No (ref)	2933 (21.6)	1
Yes	10,674 (78.4)	0.87 (0.77, 0.98)

Birth order of the child		
First (ref)	3142 (23.1)	1
2–3	4716 (34.7)	0.73 (0.23, 2.32)
4–5	3143 (23.1)	0.62 (0.19, 1.98)
≥6	2606 (19.1)	0.59 (0.18, 1.90)

Country		
Angola (ref)	2893(21.3)	1
DRC	6043(44.4)	1.37 (1.17, 1.61)
Zambia	4671(34.3)	0.79 (0.24, 2.54)

Ref: reference category.

Residence had association on CIAF, with children residing in rural areas having 26% higher odds of CIAF compared to those in urban areas (aOR = 1.26; 95% CrI: 1.08, 1.46). The odds of CIAF among children whose mothers secondary and higher educational level were 0.73 times less likely compared to those children whose mothers had no education aOR = 0.73; 95%CrI: 0.64, 0.83). Married mother was associated with lower odds of CIAF (aOR = 0.85; 95%CrI: 0.73, 0.99). Female children were less likely to be CIAF compared to male (aOR = 0.62; 95%CrI: 0.58, 0.67). Those children whose family wealth index is rich were 33.8% less likely to have CIAF compared to those from the poor households (aOR = 0.66; 95%CrI: 0.57, 0.80). Children from the households having big healthcare access facility had higher CIAF compared to its counterparts (aOR = 1.13; 95%CrI: 1.04, 1.24). Children with diarrhea (aOR = 1.28; 95%CrI: 1.15, 1.42) and those born as multiples (aOR = 2.84; 95%CrI: 2.24, 3.61) were more likely to experience CIAF.

Similarly, children delivered at health institution had lower chances of suffering from CIAF when compared with children delivered at home (aOR = 0.85; 95%CrI: 0.75, 0.97). Average-sized (aOR = 0.60; 95%CrI: 0.52, 0.70) and larger-sized children at birth (aOR = 0.41; 95%CrI: 0.35, 0.48) were less likely to be classified as CIAF than small sized birth. Children who received Vitamin A supplementation had 0.91 times (aOR = 0.91; 95%CrI: 0.83, 0.99) lower odds of being CIAF than their counterparts. Similarly, iron supplementation had lower odds of being CIAF (aOR = 0.87; 95%CrI: 0.77, 0.98).

Children with moderate, mild, and severe anemia were (aOR = 1.14; 95%CrI: 1.02, 1.27), (aOR = 1.33; 95%CI: 1.20, 1.48), and (aOR = 1.95; 95%CrI: 1.43, 2.66), respectively were less likely to be classified as CIAF than non anemic. Children from the DRC had 1.37 times (aOR = 1.37; 95%CrI: 1.17, 1.61) higher odds of developing CIAF compared to children from Angola (Table 4).

The random effects analysis based on the Besag–York–Mollié (BYM) model revealed substantial variability in the precision parameters associated with environmental, demographic, and spatial components (S2). In this study, relatively high precision estimates were observed for global footprint (posterior mean = 35,518; 95% CrI: 5811, 108,162), number of under-five children (posterior mean = 22,402; 95% CrI: 1551, 87,268), and structured spatial effects (posterior mean = 22,032; 95% CrI: 1469, 86,145), indicating lower variability and stronger contributions of these components to the model. In contrast, the precision for unstructured spatial effects (posterior mean = 57; 95% CrI: 24, 117), suggesting greater unexplained random variation at the local level which indicates there is residual heterogeneity that is not captured by the structured spatial component (S2).

## Discussion

This study was conducted in three African countries with recent DHS data from 2023 to 2024, aiming to assess the spatial distribution and pooled prevalence of the CIAF. The study found that the pooled prevalence of the CIAF in three African countries was 41.2% (95%CI: 40.4, 42.1). This result is nearly in line with study conducted in Ethiopia 40.69%) [44] and in the rural area of the Bogor District in Indonesia (42.12%) [45]. Though, higher with studies conducted in West Bengal, India 32.7% [46] and Argentina (15.1%) [47]. However, this is lower than studies conducted in Yemen 70.1% [48], Bangladesh 48.3% [26], and southern India 58.59% [49]. The variation in prevalence across these regions can be attributed to differences in socioeconomic conditions, healthcare access, and public health interventions, which influence child growth and malnutrition rates. Moreover, differences in dietary patterns, living environments, and climatic conditions may substantially contribute to the observed variation in CIAF across countries such as inadequate dietary diversity, limited access to protein-rich and micronutrient-dense foods, poor living environments characterized by overcrowding, inadequate sanitation, and unsafe water sources, rainfall variability, and temperature extremes can influence agricultural productivity, food availability, and disease patterns, ultimately affecting child nutritional status. CIAF poses amplified public health challenges, increases morbidity rates, and complicates treatment efficacy. This study highlights the need for integrated healthcare strategies; prioritize inter-sectorial and multi-country interventions, such as improved hygienic practices and other supplementation. Furthermore, policymakers should prioritize resource allocation in regions with the highest CIAF burden.

The odds of children experiencing CIAF were lower for children from rich households than for those from poor households. This finding is consistent with those of other studies [50], [51]. This could be because the wealthiest households can afford to buy different types and amounts of food for their children, improving mothers' ability to afford the cost of healthy food and ensuring household food security, while poorer homes may have less access to health care services than wealthier ones [44].

The results revealed that children living in rural areas are more likely to have CIAF than urban children. This is consistent with previous studies conducted in Myanmar [52] and Bangladesh [53]. This may be explained by differences in living environments, including limited access to clean water, sanitation, healthcare services, difficult access to food and markets in rural settings.

In this study, multiple births were more likely to have CIAF than single births, which is supported by a study conducted in Ethiopia [54]. This may be due to children from multiple births, where inadequate breastfeeding and competition for nutrition are more common [55].

The findings showed that female children had a lower risk of CIAF than male children. This study is consistent with previous studies [56], [57], [58]. This could be because male infants are biologically more vulnerable to growth and illness in early life and have a higher rate of preterm births than females [59], [60]. Additionally, there is a perception that girls are less likely to be affected by environmental stress than boys [61].

The results of this study also suggested that average- and large-weight-born children had a lower chance of CIAF compared to children with a small birth size, consistent with another study [57]. This may be because individuals with small birth size may be prone to certain infectious diseases, and their immunity may not be strong enough to fight such infectious diseases, which may lead to the development of other anemic diseases over time.

The odds of being classified as CIAF were lower for children whose mothers were educated, consistent with previous studies conducted in Ethiopia [57]. This may be due to educated mothers having adequate healthcare and nutrition knowledge, and paying more attention to child-rearing practices than uneducated mothers. Furthermore, they ensure a hygienic environment for children, possess a greater understanding of caregiving, and exhibit increased requirements for nutritious diets [62].

Moreover, this study found that anemic children were more exposed to CIAF than non-anemic children, which is in line with a study conducted in Ethiopia [56]. Similarly, vitamin A supplementation reduced the risk of CIAF, which is in line with a study conducted in Nepal [63]. This might be vitamin A supplementation improves immunity, reduces infection risk, and is associated with better growth outcomes among under-five children.

Children who experienced diarrhea were more likely to suffer from CIAF than those without recent diarrhea. This is consistent with a previous study conducted in Bangladesh [64]. This might be due to repeated diarrheal episodes of intestinal infection leading to malabsorption and nutrient loss, which adversely affects growth. Additionally, children born in health institutions faced reduced risks of CIAF, aligning with findings that institutional delivery is associated with improved neonatal care and access to child health services [65].

The findings of this study showed that women's age had a negative effect on CIAF. This finding is similar to that of previous study conducted in Nepal [63]. This might be explained by the fact that older mothers often have greater childcare experience, improved decision-making capacity, and better access to household resources, all of which can contribute to improved child feeding practices and healthcare utilization.

Our study also showed that the number of children under five years of age from households had a higher risk of CIAF. This finding is consistent with that of a previous study [56]. The possible explanation for this finding is that increased number of under-five children, resources like food, care, and attention become more stretched, making individual children more vulnerable to malnutrition.

Moreover, the findings of this study showed that child age had a positive association with CIAF, which is consistent with past study in Ethiopia [66]. This might be a lack of adequate and balanced food intake to meet the metabolic demand for childhood growth as they aged and older children's frequent interactions with their unhygienic surroundings, which may increase the risks of exposure to childhood infectious diseases such as diarrheal diseases, parasite infections and other acute illnesses.

In this study, we first analysed the linear model and extended this assumption with a more complex assumption. We also observed spatial autocorrelation in the data, which allowed us to incorporate these two properties into an appropriate model. The existence of this spatial dependency in the CIAF was tested using Moran's I and Geary's C tests of spatial autocorrelation, which confirmed the presence of spatial dependence in our data. This difference may be due to climatic variations in geographical, political, and sociocultural norms and dietary-related factors in different provinces of the countries.

Therefore, from these two separate analyses, it was found that non-linear random effects of factors associated with CIAF and spatial autocorrelations were observed in the data. To fully leverage these two properties existed in the data, we employed a Bayesian additive mixed model with a Besag-York-Mollie (BYM) mixed effect model to determine the effects of factors on CIAF and the spatial disparities of CIAF among under-fives across the three African countries.

In addition, our analysis found that nonlinear environmental parameters, such as aridity, daytime land surface temperature, and the global human footprint, all have a considerable impact on the CIAF. This could be because extreme weather conditions and vegetation differences affect population settlement, agricultural output, and access to health infrastructure; however, urbanization and human alteration present both opportunities and restrictions for health care, as they directly and indirectly affect food systems, disease exposure, and access to health services [67], [68].

## Strengths and limitations of the study

The strengths of this study were that it used nationally representative survey DHS data across multiple countries. This study applied a Bayesian geo-additive modelling approach, which allows for a comprehensive analysis of spatial disparities, and attempted to assess the non-linear effects of factors in the CIAF across three African countries, which allows us to see the cross-country disparities and CIAF. Nonetheless, the study has certain limitations, such as its dependence on self-reported data from the DHS survey, which may introduce bias due to the potential for inaccurate recall of past events such as illness episodes, feeding practices, or timing of health-related conditions, leading to potential misclassification of key variables. Second, study had a cross-sectional design, which could not show a cause-and-effect relationship between the dependent and independent variables. Furthermore, this study did not establish fundamental causal relationships. Third, despite adjustment for several covariates, residual confounding remains possible due to unmeasured factors (dietary quality, micronutrient intake, environmental exposures, and detailed morbidity history) that may be associated with both exposures and outcomes. Finally, selection bias cannot be fully excluded, as some observations were lost due to non-response or missing data. Although sampling weights were applied to improve representativeness, systematic differences between included and excluded participants may still influence the results.

## Conclusions

The overall prevalence of CIAF in the three African countries is 41.2%, based on their recent DHS survey. Although the DRC has shown the maximum CIAF at 46.0%, this is still a much higher proportion than the CIAF in developed countries. In this study, we first performed a preliminary analysis using linear regression, and this linearity was then extended to a nonlinear assumption by relaxing the effects of the factor. Moreover, the Moran's I and Gears C test of spatial dependency with different neighbourhood structures proved the existence of spatial dependence of the data. To consolidate these two properties in the data, we used the Bayesian geo-additive model with the BYM mixed effect model to determine the spatial disparities of the CIAF among children across the three African countries. The findings of this study show the existence of spatial disparities in CIAF, and the effect of risk factors is found to have a non-linear effect on CIAF across the three African countries. The higher and inequality of CIAF are influenced by the underutilization of healthcare services, economic status, women's education, coverage disparities, poor/absence of transportation facilities, and child supplementation food. The study showed that place of residence, level of education, sex of child, wealth index, marital status, access to health facility, birth size of child, type of birth, diarrhea, vitamin A supplementation, place of birth, child anemia status, iron supplementation and country have effects in the CIAF. Moreover, the identified socio-demographic characteristics and provinces at a higher risk of CIAF can be used to inform localized intervention and prevention strategies to improve children's nutritional status and health care in the study area.

## Clinical trial number

Not applicable.

## Author contribution

NAB and AAA involved in conception, design, or acquisition, data clearance and analysis, and interpretation of data; KTG and ZMW involved in data analysis. DBB and DC involved in a thorough revision for essential intellectual content. The article was read and approved by every author.

## CRediT authorship contribution statement

**Nigussie Adam Birhan:** Writing – review & editing, Writing – original draft, Validation, Software, Methodology, Investigation, Formal analysis, Data curation, Conceptualization. **Abebew Aklog Asmare:** Validation, Software, Formal analysis, Data curation, Conceptualization. **Kefale Tilahun Getahun:** Writing – original draft, Methodology. **Zelalem Meraf wolde:** Writing – original draft. **Denekew Bitew Belay:** Writing – review & editing. **Ding-Geng Chen:** Writing – review & editing, Supervision.

## Consent for publication

Not applicable.

## Ethics approval and consent to participate

This research is based on publicly available, anonymous secondary data from the DHS Program. The DHS data collection techniques were evaluated and approved by the ICF Institutional Review Board (IRB) in the United States and the Department of Health and Human Services' rules for human subject protection (45 CFR 46). All DHS surveys required informed consent, and confidentiality was rigorously protected. Participants aged ≤16 years provided parental or guardian consent. We obtained permission to use the data from the DHS Program https://dhsprogram.com/ and this secondary analysis did not require any additional ethical approval. We confirm that all methods were performed in accordance with the relevant guidelines and regulations and that this study adheres to the ethical principles of the Declaration of Helsinki.

## Funding

This research received no specific grant from any funding agency in the public, commercial, or not-for-profit sectors.

## Declaration of competing interest

The authors declare no competing interests.

## Data Availability

The data used in this study is from demographic and health survey data which can be available via reasonable request from their website at DHS repository, https://www.dhsprogram.com.

## Appendix A Supplementary data

Supplementary Material 1: Comparison of different models (GLMM, GAMM, and GGAMM) using DIC, WAIC, MLIK, and CPO information criteria.

Supplementary Material 2: Posterior estimates of random effect precision parameters in the Besag–York–Mollié (BYM) spatial model with corresponding 95% credible intervals.
