# Supplementary material for: Spatial disparities and associated factors of composite index of anthropometric failure for under-five children across three African countries
Source: Glob Epidemiol. 2026 May 25;11:100268. doi: 10.1016/j.gloepi.2026.100268 (PMC13234250; doi:10.1016/j.gloepi.2026.100268)
Supplement: Supplementary file 1 — Supplementary Material 1: Comparison of different models (GLMM, GAMM, and GGAMM) using DIC, WAIC, MLIK, and CPO information criteria. [file mmc1.docx]

S1**:** Comparison of different models using information criteria

| **No** | **Model** | **DIC** | **WAIC** | **MLIK** | **CPO** |
| --- | --- | --- | --- | --- | --- |
| 1 | GLMM | 17060 | 17151 | -8742 | -8554 |
| 2 | GAMM | 16466 | 16598 | -8579 | -8269 |
| 3 | GGAMM | 16024 | 16395 | -8389 | -8124 |
