# Supplementary material for: Spatial disparities and associated factors of composite index of anthropometric failure for under-five children across three African countries
Source: Glob Epidemiol. 2026 May 25;11:100268. doi: 10.1016/j.gloepi.2026.100268 (PMC13234250; doi:10.1016/j.gloepi.2026.100268)
Supplement: Supplementary file 2 — Supplementary Material 2: Posterior estimates of random effect precision parameters in the Besag–York–Mollié (BYM) spatial model with corresponding 95% credible intervals. [file mmc2.docx]

S2: Posterior estimates of random effect precision parameters in the BYM spatial model

| **Random effects** | **Besag (BYM)** | | |
| --- | --- | --- | --- |
| **Model Hyper parameters** | **Estimate** | **2.5%** | **97.5%** |
| Precision for Aridity | 1167 | 167 | 4207 |
| Precision for land surface temperature during the day | 432 | 48 | 1744 |
| Precision for global footprint | 35518 | 5811 | 108162 |
| Precision for current age of mother | 10116 | 301 | 49877 |
| Precision for number of under-five children | 22402 | 1551 | 87268 |
| Precision for age of child in months | 9886 | 729 | 38612 |
| Precision for structured spatial effects | 22032 | 1469 | 86145 |
| Precision for unstructured spatial effects | 55 | 24 | 117 |
